# Analysis of Healthcare Resource Utilization and Costs after the Initiation of Biologic Treatment in Patients with Ulcerative Colitis and Crohn’s Disease

**DOI:** 10.36469/9791

**Published:** 2018-09-01

**Authors:** Sue Perera, Shibing Yang, Marni Stott-Miller, Joanne Brady

**Affiliations:** 1 GlaxoSmithKline, Uxbridge, Middlesex, UK; 2 GlaxoSmithKline, Collegeville, PA, USA

**Keywords:** biologics, Crohn’s disease, healthcare costs, resource utilization, ulcerative colitis, inflammatory bowel disease, claims analysis

## Abstract

**Background:**

This retrospective cohort study aimed to describe and quantify healthcare resource utilization and costs for patients with ulcerative colitis (UC) and Crohn’s disease (CD) following initiation of biologic therapy.

**Methods:**

Resource utilization and costs were analyzed at baseline and 1- and 2-years after initiating a biologic. Data were extracted from a US administrative health insurance claims database for adults ≥18 years. Eligible patients were continuously enrolled in a health plan with medical and pharmacy benefits for ≥12 months prior to, and 12 months (primary analysis) or 24 months (secondary analysis) after index date (biologic initiation).

**Results:**

In total, 4864 and 2692 patients with UC, and 8910 and 5227 patients with CD were identified in the 1- and 2-year follow-up cohorts, respectively. Of 1-year follow-up cohort patients, 45% received the same biologic initiated at index for ≥1 year. Infliximab and adalimumab were the most commonly initiated biologics in patients with UC or CD. The highest proportion of patients who continued with the same biologic after 1- and 2-years had initiated therapy with infliximab for both indications (although at the 1-year follow-up for CD, the highest proportion continued to use natalizumab, but this was a small sample [n=15]).

Generally, the proportion of patients having inpatient admissions and emergency department (ED) visits decreased after receiving the same biologic for 1 year compared with baseline, although the proportion having outpatient visits did not change. Mean per patient all-cause costs for inpatient hospitalizations, ED visits and outpatient visits decreased for patients with UC or CD who received the same biologic for 1 year, while mean pharmacy costs per patient increased.

**Conclusions:**

This descriptive analysis shows that although biologics effectively reduced inpatient and ED resource utilization and corresponding costs in patients with UC and CD, total management costs increased, driven by increased pharmacy costs.

## Background

Ulcerative colitis (UC) and Crohn’s disease (CD) are the two main forms of inflammatory bowel disease (IBD), a chronic, idiopathic, inflammatory condition of the digestive system that disproportionately affects Western societies in the Northern hemisphere.^1^ In the United States (US), IBD affects approximately 1.7 million individuals, increasing by up to 70 000 new diagnoses per year.^1^ IBD was associated with almost 200 000 hospitalizations per annum in the US in 2010. In addition, approximately 1.9 million outpatient physician visits are required annually to manage the milder symptoms of the disease and for routine monitoring and drug administration.^2^ These trends drive an increase in inpatient, outpatient and pharmacy direct costs associated with IBD, which are estimated to be between $11 billion and $28 billion annually in the US alone.^1^,^3^ IBD also incurs indirect medical costs^4^ as patients with IBD are significantly more likely than the general population to lose days at work due to illness.^5^

The annual per-patient cost of IBD management is dependent on the disease severity and the relative frequency of disease exacerbations (flare ups) and remissions. Outpatient medical management is less costly than inpatient medical management^6^ and both are significantly less costly than elective or emergency surgery.^7^ Management strategies that can promote and maintain disease remission are therefore likely to be of significant value in terms of patient quality of life (QoL), resource utilization and the overall cost of treatment.^3^

Treatment for IBD aims to achieve clinical remission and promote mucosal healing.^1^,^8^ Current pharmacotherapies include aminoacylates, corticosteroids and immunosuppressants and may be used either alone or in combination.^9^–^11^ Biologics may be prescribed for patients who have moderate-to-severe disease, who have become refractory to standard treatment, or to induce and maintain mucosal healing and remission.^9^–^12^ Biologics used in IBD are monoclonal antibodies that inhibit various components of the pro-inflammatory cascade, typically tumor necrosis factor-alpha (TNFα), and thus prevent the chronic inflammation that underlies IBD. Since the first anti-TNFα biologic, infliximab, was approved in 1998 for CD,^13^ several other biologic agents have been approved for use in IBD treatment, including alternative anti-TNFα agents (e.g. adalimumab in UC and CD, and golimumab in UC only^14^,^15^) and biologics with other mechanisms of action, such as antibodies against interleukin (IL)-12 and IL-23 (e.g. ustekinumab in CD^16^) or integrin inhibitors (e.g. vedolizumab in UC and CD^17^). Biologics have been shown to be well tolerated^18^ and effective^19^ for the induction and maintenance of remission of UC and CD. The use of biologics can lead to a reduction in inflammatory markers,^20^ and improved QoL in patients with IBD.^21^ Furthermore, biologics have been shown to significantly reduce the risk of colectomy in patients with UC who had failed to respond to high-dose intravenous corticosteroid therapy,^22^ and to reduce the risk of hospitalization in patients with IBD.^23^

However, there are limited real-world data on healthcare resource utilization and costs for patients with IBD receiving biologic therapy. The aim of this retrospective, insurance claims-based cohort study was to describe and quantify healthcare resource utilization and all-cause costs for patients with UC and CD following the initiation of biologic therapy. These data may inform clinical trial design and economic evaluations with the aim of optimizing the medical management of patients with IBD.

## Methods

### Study design

This is a ‘new-user’ retrospective cohort study.^24^ Briefly, retrospective data were collated from patients in the US from the time of biologics initiation, to allow assessment of their pre-treatment characteristics and to capture costs and healthcare resource utilization that occurred during follow-up.^24^

### Study objectives

The primary objectives were to quantify annual healthcare resource utilization and all-cause costs for patients with UC and CD in the year before and the year after initiating a biologic: (i) in patients who continued to receive the same biologic for at least 1 year after initiation (‘as treated’ population); (ii) in all patients irrespective of whether they continued with their initiated biologic therapy (‘intention to treat’ population [ITT]). The secondary objectives were to repeat these analyses using a 2-year follow-up period after the initiation of a biologic.

### Data source and patient population

Data were obtained from administrative health insurance claims from the Truven Health Analytics* Research Databases, which included the Truven Health MarketScan Commercial and Medicare Supplemental Databases that contain de-identified healthcare data for individuals in the US. The MarketScan Commercial Database includes data for employees and their dependents who are <65 years of age and are privately insured under a variety of health plans. The MarketScan Medicare Supplemental Database includes data for retirees with Medicare supplemental insurance sponsored by their previous employers. All enrollment records and inpatient, outpatient, ancillary, and drug claims were collected. This study was exempt from Institutional Review Board review as the analysis used retrospective health insurance claims data provided in aggregate format precluding subject identification and involving no direct subject contact.

Data were extracted for adults (≥18 years of age) who initiated biologic therapy between April 1, 2010 and March 31, 2015 (primary analysis) or March 31, 2014 (secondary analysis). The biologics examined were adalimumab, certolizumab, infliximab, natalizumab, ustekinumab, golimumab, and vedolizumab. Except for vedolizumab, biologic therapy was identified from medical claims using Healthcare Common Procedure Coding System (HCPCS) or from pharmacy claims using National Drug Codes (NDC) (Supplementary Table 1). Likely vedolizumab use was identified using an algorithm as no specific HCPCS code was available prior to 2016. Specifically, vedolizumab use was identified by prescription claims for vedolizumab, claims with unclassified HCPCS code J3590 along with a primary diagnosis code for UC or CD, or claims with HCPCS codes C9026 and J3380.

**Figure 1:**
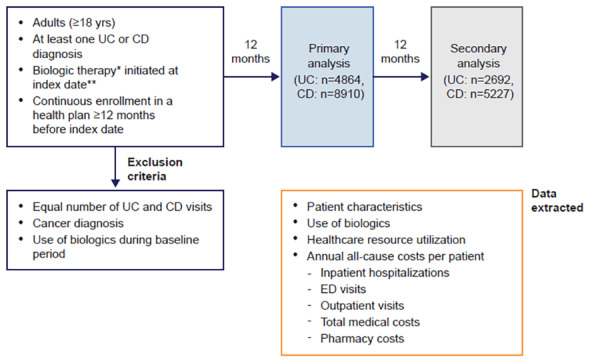
Study Design Patient population, follow-up periods and data extracted for analysis. *Adalimumab, certolizumab, infliximab, natalizumab, ustekinumab, golimumab, vedolizumab. **Between April 1, 2010 and March 31, 2015 (primary analysis) or March 31, 2014 (secondary analysis). CD: Crohn’s disease; ED: emergency department; UC: ulcerative colitis

The index date was defined as the date on which a biologic therapy was initiated. Individuals were required to have had continuous enrollment in a health plan with medical and pharmacy benefits for a minimum of 12 months prior to the index date (baseline period). Patients were required to have at least one diagnosis of UC (The International Classification of Diseases, Ninth Revision, Clinical Modification [ICD-9-CM] code 556.xx) or CD (ICD-9-CM code 555.xx; Figure 1) during the baseline period or on index date. Individuals with claims for both UC and CD were assigned to either the UC or CD cohort based on the number of healthcare visits with a UC or CD diagnosis, and patients with equal numbers of UC and CD healthcare visits were excluded (n=180). Patients who had received a biologic during the baseline period were excluded (n=5924 and 1608 for CD and UC, respectively). A minimum of 12 months of continuous enrollment after the index date was required for inclusion in the primary analysis and a minimum of 24 months of continuous enrollment was needed for inclusion in the secondary analysis. Patients who were diagnosed with cancer during the study period were excluded (n=586 and 329 for CD and UC, respectively).

### Data extracted

Data obtained from the databases included patient characteristics (age, sex, geographic region, health plan type) on the index date and the Charlson comorbidity index score^25^,^26^ during the baseline period (Figure 1).

Biologic use, including the most common therapies initiated, the time on therapy, and the proportion of patients who continued use of the initiated biologic, was described. Only the biologic with which patients initiated treatment was recorded; subsequent biologic use was not examined. Measures of healthcare resource utilization included the proportion of patients who required outpatient services (i.e. services at a clinic, outpatient hospital, or physician office), emergency department (ED) visits and inpatient hospitalizations, as well as the number of times an individual used these resources.

Annual all-cause costs per patient were reported for outpatient services, ED visits, inpatient hospitalizations, total medical costs, and pharmacy costs. Total medical costs included costs for medical services incurred in all healthcare settings, which were predominantly from outpatient visits, ED visits and inpatient visits. Total medical costs also included costs from healthcare settings such as home healthcare, hospice facility, skilled nursing facility, etc., but these are marginal and not reported separately. The pharmacy cost was calculated as the cost of medications dispensed at a pharmacy (identified using NDC codes) or administered in a healthcare facility (identified using medication HCPCS codes), which includes both biologics and non-biologics. The sum of the total medical cost and the pharmacy cost was also reported. All costs included costs paid by private or public insurance and out-of-pocket costs by patients.

### Data analysis

Cohort selection and the creation of analytic variables were undertaken using the Instant Health Data platform (Boston Health Economics, Boston, MA, USA). Statistical analyses were carried out using R, version 3.2.1 (The R Foundation for Statistical Computing, Vienna, Austria). All analyses were descriptive in nature. Analyses were conducted separately for patients with UC and CD and were stratified by the prescribed biologics. To adjust for inflation, all costs were adjusted to US$ 2015 using the Medical Care Component of the Consumer Price Index.

Analyses were conducted for the as-treated as well as the ITT populations. The as-treated population was comprised of all patients who continued to receive the same biologic prescribed at index throughout the follow-up periods, and was considered the principal population for the healthcare resource utilization and cost analyses. For the as-treated analyses, continuous use of a biologic was assumed if patients did not have a gap in therapy that exceeded a period of time that was defined based on the expected dosing period for maintenance therapy, plus a 30-day grace period, as outlined for each biologic in Supplementary Table 1. The ITT population comprised all eligible patients who initiated a biologic at index, irrespective of whether they continued to receive it or not for the duration of the follow-up period.

## Results

### Patient population

The majority (>85%) of patients initiating biologics (1- and 2-year cohorts) with UC and CD were <60 years of age (Table 1). The proportion of patients with UC was similar among the <60 years age groups in both 1-year and 2-year cohorts. The highest proportion of patients with CD were in the 18–29 years old age group for both 1- and 2-year cohorts. The proportion of males and females was similar among patients with UC, however, there was a higher proportion of females with CD who initiated biologics.

### Usage of biologics

**Figure 2:**
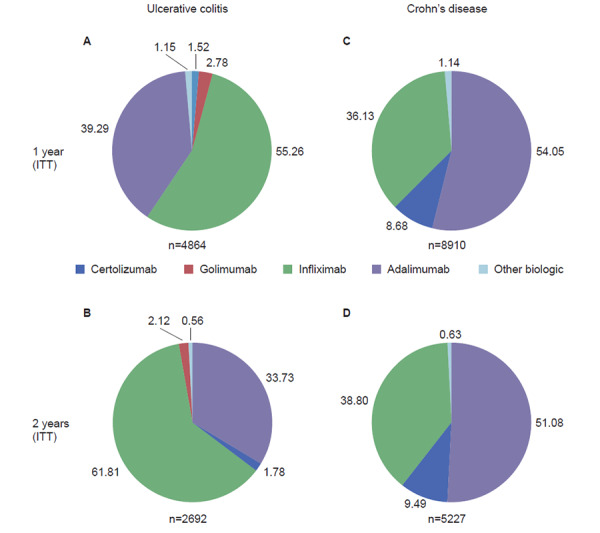
Biologics Initiated in Patients with UC and CD in the 1-year and 2-year Cohorts Biologics initiated in patients with UC (A and B) and CD (C and D) included in the 1-year (A and C) and 2-year (B and D) cohorts, irrespective of whether they continued to receive them or not during follow-up (ITT population). Values indicate percentage of patients. CD: Crohn’s disease; ITT: intention-to-treat; UC: ulcerative colitis

**Figure 3:**
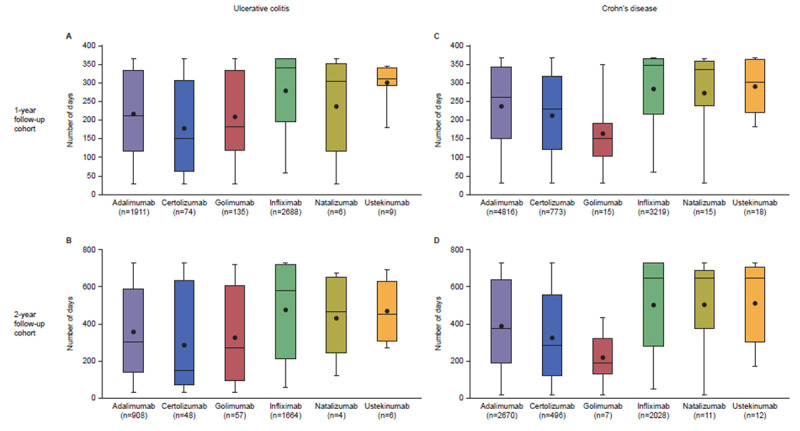
Duration of Biologic Therapy in Patients with UC and CD in 1-year and 2-year Cohorts Median duration (with interquartile range) of biologic therapy in patients with UC (A and B) and CD (C and D) in the 1-year (A, C) and 2-year follow-up (B, D) cohorts (ITT population). Mean value represented by black circle. Note: natalizumab and ustekinumab are not currently approved for treating UC and are likely captured due to off-label prescription and/or the algorithm used to categorize patients with UC versus CD. CD: Crohn’s disease; ITT: intention-to-treat; UC: ulcerative colitis

In total, there were 4864 and 2962 patients with UC, and 8910 and 5227 patients with CD who initiated biologic therapy in the 1-year and 2-year cohorts, respectively (ITT population; Figure 2). Infliximab followed by adalimumab were the most commonly initiated biologics in patients with UC in the 1- and 2-year cohorts (Figures 2A and B), while in patients with CD, adalimumab followed by infliximab were the most common (Figures 2C and D). In both the 1- and 2-year cohorts, the median time on therapy was longest for patients with UC or CD initiating infliximab (1-year cohort: 343 days and 346 days for UC and CD, respectively; 2-year cohort: 579 days and 642 days for UC and CD, respectively) (Figure 3). For patients with UC and CD receiving adalimumab, median time on therapy was 213 days and 261 days, respectively, in the 1-year cohort and 306 days and 370 days, respectively, in the 2-year cohort. The shortest median time on therapy in UC was for patients initiating certolizumab (152 days for both 1- and 2-year cohorts; Figures 3A and B), and in CD, for patients initiating golimumab (1- and 2-year cohorts: 152 and 190 days, respectively; Figures 3B and C). However, data for certolizumab and golimumab should be interpreted with caution due to the small sample sizes.

Overall, 2195/4864 (45.1%) and 4017/8910 (45.1%) of patients with UC and CD, respectively, continued to receive the same biologic initiated at index for at least 1 year (data not shown).

**Figure 4:**
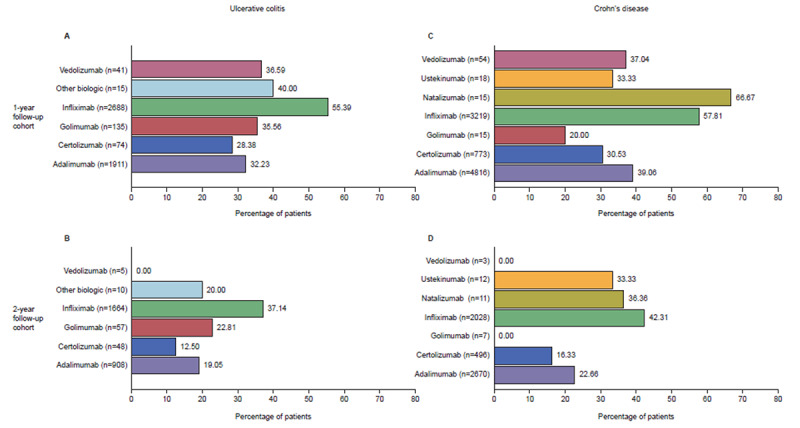
Proportion of Patients with UC and CD Who Received the Same Biologic Throughout Follow-up Proportion of patients with UC (A and B) or CD (C and D) who continued to receive the same biologic initiated throughout the follow-up period: 1 year (primary analyses; A and C) and 2 years (secondary analyses; B and D) (1- and 2-year follow-up cohorts). N numbers represent the number of patients who started a given biologic (ITT population), while the percentage shows the proportion of patients who continued receiving the same biologic over the follow-up period (as-treated population). CD: Crohn’s disease; ITT: intention-to-treat; UC: ulcerative colitis

In patients with UC, the highest proportion of those who continued to receive the same biologic for at least 1 year and 2 years had initiated therapy with infliximab (55% for 1 year - Figure 4A, 37% for 2 years - Figure 4B).

Whilst adalimumab was the second most commonly-initiated biologic in patients with UC, 32% and 19% of patients continued to receive it over the 1- and 2-years of follow-up, respectively (Figure 4A and B).

In patients with CD, the highest proportion of those who continued to receive the same biologic for at least 1 year had initiated therapy with natalizumab (67%); however, this was the smallest group (n=15), limiting interpretation. More than half (58%) of patients with CD initiating therapy with infliximab continued on this therapy for at least 1 year, while less than half of CD patients initiating therapy with adalimumab continued therapy for at least 1 year (39%; Figure 4C). The highest proportion of patients who continued to receive the same biologic for at least 2 years had initiated therapy with infliximab (42%), while only about a quarter of patients who initiated therapy with adalimumab continued to receive the same biologic for at least 2 years (23%; Figure 4D).

### Resource utilization and associated costs

Data are reported here for patients who continued to receive the biologic prescribed at index for the 1-year follow-up period (as-treated population). Data for the 2-year cohort are not reported for brevity, however they are consistent with results observed for the 1-year cohort.

#### Ulcerative colitis (1-year follow-up)

In general, for most biologics, the proportion of patients with inpatient admissions and ED visits, and the mean number of visits per patient for inpatient admissions and ED visits, decreased during follow-up compared with baseline (Table 2). While similar proportions of patients had outpatient visits at both baseline and follow-up, the mean number of outpatient visits per patient increased at follow-up for most biologics (Table 2).

For the majority of biologics, mean all-cause cost per patient for inpatient hospitalization, ED visits, outpatient visits and total medical costs decreased during follow-up compared with baseline (Table 2). In contrast, it should be noted that the mean per patient outpatient costs nearly doubled at follow-up compared with baseline for vedolizumab. However, interpretation of this observation is limited given the low sample size, large standard deviation and use of an algorithm to identify vedolizumab use. The mean pharmacy costs per patient increased between baseline and follow-up with all biologics, most likely because of biologic prescriptions. The mean combined medical and pharmacy costs per patient also increased, likely driven by all-cause pharmacy costs (Table 2).

### Crohn’s disease (1-year follow-up)

The results from the CD cohort are similar to the data observed for patients with UC (Table 3). In brief, the proportions and per patient mean all-cause costs of inpatient hospitalization and ED visits decreased at follow-up for most biologics; however, the proportion of patients with outpatient visits remained unchanged at follow-up and no clear trend was observed for mean per patient outpatient visits. Nevertheless, increased mean per-patient pharmacy costs were observed at follow-up compared with baseline for all biologics, which contributed to increased combined per patient medical and pharmacy costs.

Resource utilization for patients who initiated treatment with a biologic, irrespective of whether they continued to receive it or not (ITT analysis), are shown in Supplementary Tables 2 and 3.

## Discussion

The availability of biologics has led to a realistic goal of achieving prolonged remission for many patients with IBD. The literature suggests that use of biologic therapy has changed the profile of expenditure and healthcare resource utilization of patients with IBD, leading to higher pharmacy costs but lower healthcare resource utilization.^3^,^27^ The results of this study are in agreement with these prior observations that although biologics effectively reduced inpatient and ED healthcare resource utilization and corresponding costs, these savings are only partially offset by the increased pharmacy costs associated with biologics, leading to higher overall management costs for patients with IBD.

Several studies have investigated the trends in drug use and associated costs over recent years in patients with IBD. Rocchi *et al*^28^ analyzed private and public Canadian claims databases and found that the majority of costs associated with IBD are accounted for by medication costs, predominantly for infliximab and adalimumab. This represents a shift from a decade ago when hospitalization costs represented the largest component. Yu *et al*^29^ used the Truven MarketScan database to examine drug utilization trends, finding a rise in the market share of biologics from 2007 to 2015. The majority of costs associated with out-patient medication use were driven by increasing use of biologic therapies in patients with IBD. Rubin *et al*^30^ analyzed patients newly initiating treatment using a commercial US claims database. Frequent dose and treatment changes were observed for both patients with UC and those with CD; costs were substantially higher in patients with suboptimal treatment. Together, these studies corroborate our findings; pharmacy-related healthcare utilization represents the major cost driver in IBD, reflecting the increased use of biologic therapies, and reduced hospitalization. However, none of these studies have examined healthcare resource utilization and costs for patients newly initiating biologics; as management of IBD is increasingly relying on biologic therapy, there is a need to study the impact of biologic therapy on the economic burden of the disease.

Overall, the proportion of patients with UC or CD inpatient admissions and ED visits decreased after receiving the same biologic for 1 year, compared with the year prior to initiation, while the proportion of patients with outpatient visits remained generally the same. Despite the use of a biologic, the similarity of outpatient visits per patient in the baseline and follow-up periods could be due to patients’ regular monitoring visits and could also reflect the visits required for intravenous administration of specific biologic therapy. Despite the number of outpatient visits per patient generally remaining the same, outpatient costs per patient typically decreased once a biologic was initiated and continued for at least 1 year, suggesting that outpatient visits may have been more routine in nature and involved fewer additional procedures. Patients using vedolizumab in both UC and CD cohorts were the exception in that outpatient visits and costs appeared to increase in the follow-up period in this group. This difference could potentially reflect extra resource and costs involved in the intravenous administration of vedolizumab and/or extra monitoring due to less experience using vedolizumab. The patient population may also be different. Vedolizumab may be considered to have a favorable safety profile compared with anti-TNFs in patients for which systematic immunosuppression is best avoided, such as the elderly or patients with more comorbidities.^31^,^32^ However, vedolizumab also has a slower onset of action compared with anti-TNFs^32^,^33^ and so infliximab may be prescribed for patients with difficult-to-control IBD, before switching to vedolizumab as a long-term therapy. Alternatively, this finding may simply be an artifact of low sample size.

Although we observed increased pharmacy costs associated with the use of biologics, the reduction in hospitalizations and ED visits may have wider benefits to patients and society, such as potential reduction in the number of days off work in patients with IBD and overall productivity. In addition, this study does not account for the improved QoL in patients and the positive impact on mortality attributed to the use of biologics. LeBlanc *et al* (2015, Cochrane report) concluded that biologics improved the QoL of patients with UC.^21^ In a meta-analysis and systematic review, biologics were shown to deliver improved rates of disease regression^19^ and hospitalization,^23^ and reduced surgical intervention versus placebo in a randomized trial.^22^ Therefore, an understanding of all these aspects of patient management are necessary for a more holistic evaluation of the overall value of biologic therapy for patients with IBD.

In the present study, infliximab and adalimumab were the most commonly initiated biologics across all cohorts. In both patients with UC or CD, those patients initiating infliximab were the most likely to continue with therapy (more than half of patients continued to receive it beyond the first year) and achieved the longest median time on a single biologic. In the combined group of patients with UC and CD receiving adalimumab, a smaller proportion of patients continued to receive it over the 1-year (37%) and 2-year (22%) follow-up periods, respectively. These retention rates are lower than those reported in patients with IBD receiving adalimumab in other studies, which were 60% or greater at 1 year.^34^–^36^ Female sex, perianal disease, and previous infliximab use were independent predictors of treatment discontinuation; women were also more likely than men to discontinue adalimumab due to side effects.^35^ However, in the present study, the reasons for discontinuation cannot be understood.

This study has several limitations. Firstly, this was a descriptive study undertaken to address gaps in knowledge regarding healthcare resource utilization and costs in patients receiving biologic therapy for UC and CD. Thus, the analyses were not controlled for factors related to disease severity, comorbid disease, environmental factors, or age – factors that are known to influence the costs of managing patients with IBD. A retrospective study of medical records from over 1000 patients with IBD found that psychiatric illness, anemia, use of comedications (corticosteroids, narcotics) and IBD-related hospitalizations were all predictive of high treatment costs.^37^ Secondly, biologics prescribed for IBD can also be prescribed for other immuno-inflammation-related diseases such as rheumatoid arthritis, psoriasis and psoriatic arthritis, which were not excluded in the present analysis, therefore such comorbid conditions may also have contributed to the overall results. Thirdly, these analyses were limited to data from patients newly initiating biologics, or who re-started biologics after at least 1 year of non-use, indicated by no biologic use in the baseline period. Therefore, data regarding the resource utilization and costs for patients who switched biologics after less than a year of non-use were not captured. These patients may have more severe disease or may have been diagnosed with IBD for longer and this limits the generalizability of our findings. Similarly, this study only examined the biologic with which the patient initiated therapy, and subsequent biologic use was not analyzed. Further, biologics may be prescribed alone or in combination with other treatments, for example with immunosuppressants, as recommended for CD.^11^ Data on combination therapy were not captured; thus, differences in resource utilization and costs between combination therapies were not examined. The reasons why patients may have stopped or started therapies were also not known. It is important to note that research using insurance claims data has several limitations. Claims data are dependent on diagnostic coding recorded by physicians to support reimbursement. Diagnoses may be coded incorrectly or not coded at all, thereby potentially introducing measurement error. In addition, medication claims reflect the dispensed medication, but not necessarily the medication actually taken by the patient. Moreover, these results may not be generalizable to all patients with IBD using biologic therapies, including those who receive healthcare through Medicare alone or Medicaid or who do not have health insurance. Finally, this study was not designed to compare different biologics. Given the very low numbers of patients with UC or CD who could be followed-up for 2 years, with several of the biologics, particularly vedolizumab (due to its approval for IBD in 2014 and lack of specific codes until 2016), it was not possible to draw any conclusions about the long-term use of these biologics.

## Conclusions

This large, retrospective claims data analysis described real-world cost and healthcare resource utilization of patients receiving biologic therapy for UC and CD in the USA. Specifically, this analysis included only patients newly initiating biologic therapy and examined the impact of their first biologic on resource utilization and costs. The most commonly initiated biologics were infliximab and adalimumab; almost half of patients continued to receive their initiated biologic for at least 1 year, whereas the other half discontinued their initiated biologic within a year. Total resource utilization generally decreased in patients with UC or CD who received a biologic for 1 year, driven by reduced inpatient and ED visits. Consistently, total inpatient, ED and outpatient costs per patient decreased after initiating biologics, indicating that biologics may have reduced UC or CD symptomatology or reduced the frequency or severity of exacerbations. Despite the reduced resource utilization and associated costs observed after initiating biologics, total costs nonetheless increased, driven by increased pharmacy costs associated with biologic prescriptions. Further studies are needed to examine the effect of combination therapies and the switching of biologics on healthcare utilization and costs. In addition, future work should incorporate indirect management costs of patients with IBD, including costs associated with loss of productivity and absenteeism, and evaluate the improved QoL in patients receiving biologic therapy to understand holistically the economic impact and value of biologics to patients and healthcare systems.

## Supplementary Information



## Figures and Tables

**Table 1:** Characteristics of Patients with UC or CD Who Initiated Biologics (numbers describe all evaluable patients [ITT], regardless of their continued use of biologics)

		Biologic initiators with UC		Biologic initiators with CD	
		1-year follow-up, n (%)	2-year follow-up, n (%)	1-year follow-up, n (%)	2-year follow-up, n (%)
**N**		4864	2692	8910	5227
**Age, years**	18–29	1036 (21.30)	492 (18.28)	2402 (26.96)	1334 (25.52)
	30–39	1043 (21.44)	575 (21.36)	1882 (21.12)	1088 (20.82)
	40–49	1048 (21.55)	622 (23.11)	1839 (20.64)	1132 (21.66)
	50–59	1038 (21.34)	620 (23.03)	1725 (19.36)	1064 (20.36)
	60–69	516 (10.61)	275 (10.22)	807 (9.06)	457 (8.74)
	70–79	149 (3.06)	90 (3.34)	210 (2.36)	126 (2.41)
	80+	34 (0.70)	18 (0.67)	45 (0.51)	26 (0.50)
**Sex**	Female	2396 (49.26)	1347 (50.04)	4947 (55.52)	2899 (55.46)
	Male	2468 (50.74)	1345 (49.96)	3963 (44.48)	2328 (44.54)
**Region**	Midwest	1214 (25.21)	685 (25.60)	2302 (26.12)	1402 (27.05)
	Northeast	1022 (21.22)	562 (21.00)	1820 (20.65)	1014 (19.56)
	South	1762 (36.59)	976 (36.47)	3353 (38.04)	1966 (37.92)
	West	817 (16.97)	453 (16.93)	1339 (15.19)	802 (15.47)
**Charlson score**	0	3662 (75.29)	2034 (75.56)	6768 (75.96)	4042 (77.33)
	1	692 (14.23)	379 (14.08)	1202 (13.49)	668 (12.78)
	2	359 (7.38)	204 (7.58)	685 (7.69)	392 (7.50)
	3	96 (1.97)	50 (1.86)	167 (1.87)	84 (1.61)
	≥4	55 (1.13)	25 (0.93)	88 (0.99)	41 (0.78)
**Index date**	2010	561 (11.53)	445 (16.53)	1307 (14.67)	1039 (19.88)
	2011	935 (19.22)	609 (22.62)	1983 (22.26)	1328 (25.41)
	2012	951 (19.55)	714 (26.52)	1833 (20.57)	1385 (26.50)
	2013	1022 (21.01)	679 (25.22)	1687 (18.93)	1106 (21.16)
	2014	1093 (22.47)	245 (9.10)	1670 (18.74)	369 (7.06)
	2015	302 (6.21)	–	430 (4.83)	–

CD: Crohn’s disease; ITT: intention-to-treat; UC: ulcerative colitis

**Table 2:** Baseline and follow-up healthcare resource utilization and costs for patients with UC who initiated a biologic and continued to receive it for 1 year of follow-up (as-treated population). Outpatient utilization comprised physician’s office visits and outpatient hospital and clinic visits. Pharmacy costs included costs of medication dispensed at pharmacy and administered inside the healthcare facility. Total medical costs were predominantly the sum of inpatient, ED and outpatient costs.^a^

	Biologic						
	Adalimumab n=616	Certolizumab n=21	Golimumab n=48	Infliximab n=1489	Natalizumab n=3	Ustekinumab^b^ n=3	Vedolizumab^c^ n=15
**Healthcare resource utilization**							
Patients with an inpatient hospitalization, n (%)							
Baseline	126 (20.46)	4 (19.05)	11 (22.92)	504 (33.85)	1 (33.33)	0 (0)	1 (6.67)
Follow-up	50 (8.12)	1 (4.76)	2 (4.17)	172 (11.55)	0 (0)	1 (33.33)	0 (0)
Inpatient hospitalizations per patient, mean (SD)							
Baseline	0.28 (0.62)	0.24 (0.54)	0.25 (0.48)	0.47 (0.79)	0.33 (0.58)	0 (0)	0.07 (0.26)
Follow-up	0.10 (0.37)	0.05 (0.22)	0.06 (0.32)	0.15 (0.46)	0 (0)	0.33 (0.58)	0 (0)
Patients with an ED visit, n (%)							
Baseline	197 (31.98)	6 (28.57)	10 (20.83)	638 (42.85)	1 (33.33)	0 (0)	2 (13.33)
Follow-up	133 (21.59)	5 (23.81)	9 (18.75)	381 (25.59)	0 (0)	1 (33.33)	2 (13.33)
ED visits per patient, mean (SD)							
Baseline	0.55 (1.01)	0.52 (0.98)	0.27 (0.57)	0.72 (1.08)	0.33 (0.58)	0 (0)	0.27 (0.80)
Follow-up	0.28 (0.63)	0.43 (0.93)	0.19 (0.39)	0.38 (0.82)	0 (0)	0.33 (0.58)	0.20 (0.56)
Patients with outpatient visit, n (%)							
Baseline	615 (99.84)	21 (100)	48 (100)	1483 (99.60)	3 (100)	3 (100)	15 (100)
Follow-up	613 (99.51)	21 (100)	48 (100)	1486 (99.80)	3 (100)	3 (100)	15 (100)
Outpatient visits per patient, mean (SD)							
Baseline	15.84 (10.48)	15.52 (12.79)	15.10 (11.66)	17.22 (12.52)	23.00 (12.53)	16.67 (6.66)	19.87 (10.60)
Follow-up	15.35 (12.16)	19.43 (16.19)	15.29 (13.14)	22.62 (12.56)	32.00 (19.97)	18.33 (9.45)	26 (10.60)
**Costs, US$ 2015, mean (SD)**							
Inpatient hospitalization costs per patient							
Baseline	5237.16 (22 173.03)	2572.62 (5790.60)	23 005.11 (140 362.91)	9777.73 (25 098.06)	2932.86 (5079.87)	0 (0)	865.41 (3351.72)
Follow-up	1556.97 (6890.84)	482.80 (2212.46)	729.87 (3800.02)	3283.48 (20 105.26)	0 (0)	2336.22 (4046.44)	0 (0)
ED visit costs per patient							
Baseline	1038.43 (2343.31)	780.88 (1975.45)	490.31 (1365.25)	1400.01 (3427.13)	6829.45 (11 828.95)	0 (0)	369.12 (999.17)
Follow-up	600.47 (1749.04)	1528.11 (3226.87)	351.29 (903.86)	764.27 (2324.72)	0 (0)	253.86 (439.70)	282.35 (943.82)
Outpatient visit costs per patient							
Baseline	6958.17 (12 328.91)	7674.21 (9235.38)	5884.37 (7294.03)	10 125.67 (19 252.36)	31 379.25 (19 129.65)	4916.36 (2414.85)	16 312.07 (21 577.52)
Follow-up	4627.61 (6811.83)	6128.68 (6785.56)	3746.75 (4496.06)	9033.33 (12 980.08)	12 401.00 (2447.28)	2645.23 (2211.82)	34 620.88 (28 022.13)
Total medical costs per patient							
Baseline	15 092.62 (29 553.30)	12 276.04 (11 549.75)	31 145.25 (142 309.15)	23 174.47 (32 421.13)	41 152.04 (15 789.84)	5276.74 (2135.49)	18 866.09 (22 377.73)
Follow-up	7774.36 (11 875.97)	9022.00 (8764.70)	5552.71 (7653.54)	14 522.95 (26 152.63)	12 559.75 (2518.88)	5323.34 (5959.76)	36 653.80 (30 519.07)
Pharmacy costs per patient							
Baseline	7656.26 (7809.05)	7435.10 (6597.44)	7738.25 (6266.00)	6214.10 (6675.97)	29 617.14 (12 089.04)	2237.84 (3077.25)	7942.91 (6995.66)
Follow-up	48 591.53 (16 081.84)	38 276.48 (9162.45)	55 948.19 (11 963.48)	45 710.70 (30 751.68)	76 979.14 (16 852.84)	53 905.11 (20 863.60)	35 620.01 (54 465.94)
Medical and pharmacy costs per patient							
Baseline	22 748.88 (30 289.32)	19 711.14 (13 912.50)	38 883.50 (142 544.70)	29 388.58 (33 327.49)	70 769.18 (10 588.51)	7514.57 (5168.32)	26 809.01 (22 351.87)
Follow-up	56 365.90 (21 357.74)	47 298.48 (14 953.62)	61 500.90 (14 444.79)	60 233.64 (40 682.34)	89 538.89 (19 025.45)	59 228.45 (25 054.55)	72 273.81 (46 912.99)

a Total medical costs also included a small amount of costs from healthcare settings such as home healthcare, hospice facility, skilled nursing facility, etc., these are not reported separately;

b a total of 6 patients with UC received ustekinumab or natalizumab, which are currently not approved for the treatment of UC;

c likely vedolizumab use was identified by prescription claims for vedolizumab, claims with unclassified HCPCS code J3590 along with a primary diagnosis code for UC or CD, or claims with HCPCS codes C9026 and J3380.

CD, Crohn’s disease; ED, emergency department; HCPCS; Healthcare Common Procedure Coding System; SD, standard deviation; UC, ulcerative colitis.

**Table 3:** Baseline and follow-up healthcare resource utilization and costs for patients with CD who initiated a biologic and continued to receive it for 1 year of follow-up (as-treated population). Outpatient utilization comprised physician’s office visits and outpatient hospital and clinic visits. Pharmacy costs included costs of medication dispensed at pharmacy and administered inside the healthcare facility. Total medical costs were predominantly the sum of inpatient, ED and outpatient costs^a^

	Biologic						
	Adalimumab n=1881	Certolizumab n=236	Golimumab n=3	Infliximab n=1861	Natalizumab n=10	Ustekinumab n=6	Vedolizumab^b^ n=20
**Healthcare resource utilization**							
Patients with an inpatient hospitalization, n (%)							
Baseline	570 (30.30)	73 (30.93)	0 (0)	613 (32.94)	3 (30.00)	3 (50.00)	10 (50.00)
Follow-up	275 (14.62)	54 (22.88)	0 (0)	263 (14.13)	0 (0)	0 (0)	3 (15.00)
Inpatient hospitalizations per patient, mean (SD)							
Baseline	0.43 (0.78)	0.49 (0.89)	0 (0)	0.49 (0.88)	0.30 (0.48)	1.67 (2.25)	0.75 (0.85)
Follow-up	0.20 (0.56)	0.38 (0.85)	0 (0)	0.19 (0.56)	0 (0)	0 (0)	0.20 (0.52)
Patients with an ED visit, n (%)							
Baseline	775 (41.20)	103 (43.64)	1 (33.33)	776 (41.70)	5 (50.00)	5 (83.33)	7 (35.00)
Follow-up	513 (27.27)	71 (30.09)	0 (0)	534 (28.69)	2 (20.00)	4 (66.67)	8 (40.00)
ED visits per patient, mean (SD)							
Baseline	0.70 (1.11)	0.97 (2.70)	0.33 (0.58)	0.79 (1.33)	0.60 (0.70)	2.67 (2.16)	0.70 (1.13)
Follow-up	0.48 (1.15)	0.72 (3.31)	0 (0)	0.47 (1.00)	0.30 (0.67)	0.83 (0.75)	0.60 (0.82)
Patients with an outpatient visit, n (%)							
Baseline	1877 (99.79)	236 (100)	3 (100)	1849 (99.36)	10 (100)	6 (100)	20 (100)
Follow-up	1868 (99.31)	233 (98.73)	3 (100)	1861(100)	10 (100)	6 (100)	20 (100)
Outpatient visits per patient, mean (SD)							
Baseline	16.14 (11.73)	17.80 (12.75)	27.00 (17.44)	17.27 (12.84)	19.60 (9.88)	28.00 (15.63)	26.30 (22.25)
Follow-up	15.05 (13.00)	17.67 (13.39)	21.33 (7.09)	21.94 (13.23)	30.10 (13.53)	36.83 (27.65)	31.05 (22.45)
**Costs, US$ 2015, mean (SD)**							
Inpatient hospitalization costs per patient							
Baseline	8894.42 (26 648.77)	10 492.38 (29 166.85)	0 (0)	11 683.65 (35 928.48)	11 534.97 (18 983.79)	147 170.98 (285 747.82)	12 223.66 (14 058.01)
Follow up	4199.85 (17 030.25)	8080.19 (20 565.86)	0 (0)	3918.12 (17 834.50)	0 (0)	0 (0)	2364.72 (9008.96)
ED visits costs per patient							
Baseline	1274.60 (2779.19)	1567.64 (3762.28)	1342.98 (2326.11)	1440.24 (3560.70)	727.93 (1028.85)	3569.68 (3046.72)	843.65 (1465.20)
Follow-up	1044.72 (4028.97)	1170.06 (4209.37)	0 (0)	847.99 (2368.77)	835.27 (1761.05)	2093.74 (2450.65)	1701.89 (4383.03)
Outpatient visits costs per patient							
Baseline	7803.99 (12 149.21)	9005.12 (14 665.55)	8373.34 (6598.27)	17 394.02 (29 709.42)	14 632.14 (19 283.66)	29 120.35 (11 429.60)	12 596.98 (14 273.08)
Follow-up	4521.96 (5521.87)	5303.72 (6673.90)	9391.97 (6967.49)	10 070.37 (16 781.91)	10 592.61 (4679.90)	7828.56 (8860.66)	31 374.63 (20 248.29)
Total medical costs per patient							
Baseline	19 837.11 (32 052.82)	23 518.26 (39 755.76)	18 411.21 (21 619.98)	32 576.31 (46 598.06)	27 805.68 (23 835.03)	194 206.71 (306 934.63)	26 778.24 (21 696.95)
Follow-up	10 984.94 (21 216.69)	15 960.54 (24 596.59)	9391.97 (6967.49)	16 073.35 (26 619.55)	12 653.75 (7669.55)	10 614.10 (10 895.98)	37 013.93 (22 603.98)
Pharmacy costs per patient							
Baseline	4798.63 (5700.39)	6086.57 (9669.60)	11 828.47 (8887.83)	4162.05 (5015.65)	23 474.93 (18 983.13)	8210.59 (9432.24)	7606.23 (12 849.52)
Follow-up	42 141.90 (12 709.15)	36 211.70 (12 773.02)	53 677.88 (20 737.84)	42 504.25 (33 689.20)	73 353.16 (41 617.06)	116 161.44 (40 545.36)	44 250.79 (44 740.47)
Medical and pharmacy costs per patient							
Baseline	24 635.74 (32 763.09)	29 604.84 (42 484.50)	30 239.68 (13 482.39)	36 738.35 (47 014.77)	51 280.61 (18 410.06)	202 417.30 (315 989.78)	34 384.47 (29 555.64)
Follow-up	53 126.84 (25 940.69)	52 172.23 (28 908.94)	63 069.84 (27 048.67)	58 577.60 (43 762.09)	86 006.90 (47 308.21)	126 775.54 (43 885.33)	81 264.72 (45 114.80)

a Total medical costs also included a small amount of costs from healthcare settings such as home healthcare, hospice facility, skilled nursing facility, etc., these are not reported separately;

b likely vedolizumab use was identified by prescription claims for vedolizumab, claims with unclassified HCPCS code J3590 along with a primary diagnosis code for UC or CD, or claims with HCPCS codes C9026 and J3380.

CD, Crohn’s disease; ED, emergency department; HCPCS; Healthcare Common Procedure Coding System; SD, standard deviation; UC, ulcerative colitis.

## Footnotes

* Truven Health Analytics is part of the IBM Watson Health business; MarketScan is a registered trademark of Truven Health Analytics.
