# Supplementary material for: Analysis of Healthcare Resource Utilization and Costs after the Initiation of Biologic Treatment in Patients with Ulcerative Colitis and Crohn’s Disease
Source: J Health Econ Outcomes Res. 2018 Sep 1;6(1):96–112. doi: 10.36469/9791 (PMC7309948; doi:10.36469/9791)
Supplement: Supplementary file 1 [file jheor-6-1-9791-s01.pdf]

JHEOR Journal of Health Economics and Outcomes Research

OPEN ACCESS

Supplementary Online Content

Perera S, Yang S, Stott-Miller M, *et al*. Analysis of healthcare resource utilization and costs after the initiation of biologic treatment in patients with ulcerative colitis and Crohn's disease. *JHEOR*. 2018;6(1):96-112

Supplementary Table 1. Biologics explored in the study with the HCPCS codes and NDC used to identify relevant records. Other HCPS codes used included S9359 (home use of TNFα inhibitor). As the specific HCPCS code for vedolizumab was not officially active until 2016, vedolizumab use was identified by prescription claims for vedolizumab, claims with unclassified HCPCS code J3590 along with a primary diagnosis code for UC or CD, or claims with HCPCS codes C9026 or J3380. The allowable gap was defined as the expected dosing period for maintenance therapy in IBD, plus 30 days as a grace period, and then rounded to the nearest of 45, 60, 90, or 120 days.

Supplementary Table 2. Baseline and follow-up healthcare resource utilization and costs for patients with ulcerative colitis who initiated a biologic, 1-year follow-up (intention-to-treat population).

Supplementary Table 3. Baseline and follow-up healthcare resource utilization and costs for patients with Crohn's disease who initiated a biologic (intention-to-treat population), 1 year follow-up.

This supplementary material has been provided by the authors to give readers additional information about their work.

Supplementary Table 1. Biologics explored in the study with the HCPCS codes and NDC used to identify relevant records.

Other HCPS codes used included S9359 (home use of TNFα inhibitor). As the specific HCPCS code for vedolizumab was not officially active until 2016, vedolizumab use was identified by prescription claims for vedolizumab, claims with unclassified HCPCS code J3590 along with a primary diagnosis code for UC or CD, or claims with HCPCS codes C9026 or J3380. The allowable gap was defined as the expected dosing period for maintenance therapy in IBD, plus 30 days as a grace period, and then rounded to the nearest of 45, 60, 90, or 120 days.

Therapy (HCPCS codes)	HCPCS codes	NDC	Class	Allowable gap in therapy	Expected dosing for maintenance
Adalimumab	J0135	54569552400, 74433906, 54868482200, 74379902, 74634702, 74937402, 74379903, 74379906, 74433902, 74433907	TNFα inhibitor	60 days	Every 2 weeks
Certolizumab	C9249, J0717, J0718	50474070062, 50474071079, 50474071081	TNFα inhibitor	60 days	Every 4 weeks
Golimumab	J1602	57894035001, 57894007002, 57894007102, 57894007001, 57894007101	TNFα inhibitor	60 days	Every 4 weeks
Infliximab	J1745	57894003001	TNFα inhibitor	90 days	Every 8 weeks
Natalizumab	C9126, J2323, Q4079	59075073015, 64406000801	α4-integrin	60 days	Every 4 weeks
Ustekinumab	C9261, J3357	57894006003, 57894006103, 57894006002	IL-12, IL-23 inhibitor	120 days	Every 12 weeks
Vedolizumab	J3590, C9026, J3380	64764030020	Integrinα4β7 (LPAM-1)	90 days	Every 8 weeks

CD, Crohn's disease; HCPS, Healthcare Common Procedure Coding System; IBD, inflammatory bowel disease; IL, interleukin; NDC, National Drug Codes; TNF, tumor necrosis factor; UC, ulcerative colitis

Supplementary Table 2. Baseline and follow-up healthcare resource utilization and costs for patients with ulcerative colitis who initiated a biologic, 1-year follow-up (intention-to-treat population).

Outpatient utilization comprised physician's office visits and outpatient hospital and clinic visits. Pharmacy costs included costs on medication dispensed at pharmacy and administered inside the healthcare facility. Total medical costs were predominantly the sum of inpatient, ED and outpatient costs.[a]

	Biologic (n=4864)						
	**Adalimumab n=1911**	**Certolizumab n=74**	**Golimumab n=135**	**Infliximab n=2688**	**Natalizumab n=6**	**Ustekinumab n=9**	**Vedolizumab[b] n=41**
**Healthcare utilization**							
Patients with an inpatient hospitalization, n (%)							
Baseline	456 (23.86)	15 (20.27)	29 (21.48)	1002 (37.28)	2 (33.33)	0 (0)	11 (26.83)
Follow-up	385 (20.15)	18 (24.32)	24 (17.78)	635 (23.62)	1 (16.67)	2 (22.22)	9 (21.95)
Inpatient hospitalizations per patient, mean (SD)							
Baseline	0.34 (0.74)	0.28 (0.65)	0.25 (0.51)	0.56 (0.97)	0.33 (0.52)	0 (0)	0.39 (0.74)
Follow-up	0.36 (0.91)	0.49 (1.09)	0.28 (0.77)	0.45 (1.05)	0.17 (0.41)	0.67 (1.66)	0.44 (1.05)
Patients with an ED visit, n (%)							
Baseline	678 (35.48)	25 (33.78)	34 (25.19)	1209 (44.98)	2 (33.33)	0 (0)	14 (34.15)
Follow-up	590 (30.87)	28 (37.84)	42 (31.11)	911 (33.89)	1 (16.67)	2 (22.22)	13 (31.71)
ED visits per patient, mean (SD)							
Baseline	0.63 (1.19)	0.70 (1.38)	0.40 (0.82)	0.84 (1.40)	0.33 (0.52)	0 (0)	0.73 (1.45)
Follow-up	0.58 (1.50)	0.68 (1.23)	0.45 (0.81)	0.64 (1.33)	0.17 (0.41)	0.22 (0.44)	0.66 (1.32)
Patients with an outpatient visit, n (%)							
Baseline	1910 (99.95)	74 (100)	135 (100)	2671 (99.37)	6 (100)	9 (100)	41 (100)
Follow-up	1896 (99.22)	74 (100)	134 (99.26)	2685 (99.89)	6 (100)	9 (100)	41 (100)
Outpatient visits per patient, mean (SD)							
Baseline	17.41 (12.03)	18.82 (14.88)	18.94 (14.69)	17.59 (12.77)	22.83 (10.42)	17.33 (7.84)	21.27 (18.08)
Follow-up	18.24 (13.84)	20.58 (14.79)	18.79 (16.67)	22.91 (13.87)	24.50 (15.28)	15.78 (8.50)	26.61 (16.75)

[a] Total medical costs also included a small amount of costs from healthcare settings such as home healthcare, hospice facility, skilled nursing facility, etc., these are not reported separately; [b] Likely vedolizumab use was identified by prescription claims for vedolizumab, claims with unclassified HCPCS code J3590 along with a primary diagnosis code for UC or CD, or claims with HCPCS codes C9026 and J3380.

CD, Crohn's disease; ED, emergency department; HCPS, Healthcare Common Procedure Coding System; SD, standard deviation; UC, ulcerative colitis

Supplementary Table 2 - continued. Baseline and Follow-up Healthcare Resource Utilization and Costs for Patients with Ulcerative Colitis Who Initiated a Biologic, 1-year Follow-up (intention-to-treat population)

Outpatient utilization comprised physician's office visits and outpatient hospital and clinic visits. Pharmacy costs included costs on medication dispensed at pharmacy and administered inside the healthcare facility. Total medical costs were predominantly the sum of inpatient, ED and outpatient costs[a].

	Biologic (n=4864)						
	**Adalimumab n=1911**	**Certolizumab n=74**	**Golimumab n=135**	**Infliximab n=2688**	**Natalizumab n=6**	**Ustekinumab n=9**	**Vedolizumab[b] n=41**
**Costs, US$ 2015, mean (SD)**							
Inpatient hospitalization costs per patient							
Baseline	5942.41 (18 868.39)	5605.83 (19 880.64)	11 967.70 (84 837.67)	11 163.29 (27 324.61)	2360.38 (3814.61)	0 (0)	8746.74 (25 609.51)
Follow-up	9215.80 (29 197.04)	9032.10 (27 420.72)	9573.61 (35 653.97)	13 198.47 (43 661.12)	2069.15 (5068.36)	16 773.94 (47 749.86)	12 348.36 (32 963.23)
ED visits costs per patient							
Baseline	1228.98 (3020.40)	1834.59 (5912.23)	933.07 (2221.85)	1533.48 (4268.90)	3482.39 (8332.77)	0 (0)	1876.79 (5256.10)
Follow-up	1176.61 (3606.18)	1164.49 (2361.87)	792.64 (1690.21)	1185.54 (3408.95)	30.60 (74.96)	375.31 (877.27)	1200.51 (2344.34)
Outpatient visits costs per patient							
Baseline	7971.50 (14 246.31)	8667.50 (12 558.72)	7835.79 (10 105.12)	10 067.16 (18 115.67)	19 980.84 (17 427.88)	3943.11 (3021.90)	19 345.35 (52 679.82)
Follow-up	7336.50 (12 787.62)	6320.27 (6466.33)	7092.52 (12 545.70)	10 394.35 (14 101.47)	8041.03 (5021.64)	3629.22 (5416.50)	30 226.28 (21 802.15)
Total medical costs per patient							
Baseline	16 979.77 (26 849.89)	17 652.24 (29 939.58)	22 780.83 (87 337.16)	24 672.98 (33 964.72)	25 916.75 (19 668.32)	5027.85 (4006.47)	35 172.92 (71 954.90)
Follow-up	19 254.72 (36 518.28)	17 480.67 (29 179.74)	19 835.57 (45 714.23)	26 745.64 (49 443.35)	10 281.33 (5537.16)	21 576.96 (53 959.42)	46 289.13 (43 159.30)
Pharmacy costs per patient							
Baseline	7447.76(7592.38)	8481.81 (7530.40)	8817.16 (8336.95)	6150.37 (6795.25)	25 100.68 (23 579.09)	6113.41 (6046.94)	8235.50 (6915.61)
Follow-up	41 657.03 (20 528.90)	27 859.05 (14 357.67)	48 787.23 (18 300.31)	38 402.78 (28 316.70)	48 596.82 (36 566.05)	49 682.48 (24 095.31)	24 214.55 (37 885.03)
Medical and pharmacy costs per patient							
Baseline	24 427.53 (28 077.97)	26 134.06 (31 840.37)	31 597.99 (87 992.46)	30 823.35 (34 910.93)	51 017.42 (33 361.62)	11 141.25 (7393.61)	43 408.43 (72 200.36)
Follow-up	60 911.75 (39 499.56)	45 339.72 (30 257.19)	68 622.80 (49 681.66)	65 148.42 (54 320.13)	58 878.15 (41 248.98)	71 259.44 (48 331.32)	70 503.67 (50 060.89)

[a]Total medical costs also included a small amount of costs from healthcare settings such as home healthcare, hospice facility, skilled nursing facility, etc.; these are not reported separately.
[b]Likely vedolizumab use was identified by prescription claims for vedolizumab, claims with unclassified HCPCS code J3590 along with a primary diagnosis code for UC or CD, or claims with HCPCS codes C9026 and J3380.
CD: Crohn's disease; ED: emergency department; HCPCS: Healthcare Common Procedure Coding System; SD: standard deviation; UC: ulcerative colitis.

Supplementary Table 3. Baseline and Follow-up Healthcare Resource Utilization and Costs for Patients with Crohn's Disease Who Initiated a Biologic, 1-year Follow-up (intention-to-treat population)

Outpatient utilization comprised physician's office visits and outpatient hospital and clinic visits. Pharmacy costs included costs on medication dispensed at pharmacy and administered inside the healthcare facility. Total medical costs were predominantly the sum of inpatient, ED and outpatient costs.[a]

	Biologics Initiated (n=8910)						
	**Adalimumab n=4816**	**Certolizumab n=773**	**Golimumab n=15**	**Infliximab n=3219**	**Natalizumab n=15**	**Ustekinumab n=18**	**Vedolizumab[b] n=54**
**Healthcare utilization**							
Patients with an inpatient hospitalization, n (%)							
Baseline	1529 (31.75)	248 (32.08)	1 (6.67)	1087 (33.77)	4 (26.67)	8 (44.44)	19 (35.19)
Follow-up	1095 (22.74)	219 (28.33)	3 (20.00)	680 (21.13)	0 (0)	0 (0)	15 (27.78)
Inpatient hospitalizations per patient, mean (SD)							
Baseline	0.47 (0.88)	0.55 (1.15)	0.07 (0.26)	0.52 (0.93)	0.27 (0.46)	0.89 (1.45)	0.56 (0.84)
Follow-up	0.38 (0.90)	0.50 (1.18)	0.20 (0.41)	0.34 (0.86)	0 (0)	0 (0)	0.50 (1.08)
Patients with an ED visit, n (%)							
Baseline	2141 (44.46)	345 (44.63)	4 (26.67)	1415 (43.96)	6 (40.00)	11 (61.11)	21 (38.89)
Follow-up	1667 (34.61)	295 (38.16)	3 (20.00)	1160 (36.04)	3 (20.00)	4 (22.22)	20 (37.04)
ED visits per patient, mean (SD)							
Baseline	0.85 (1.56)	0.97 (2.03)	0.33 (0.62)	0.88 (1.46)	0.53 (0.74)	1.50 (1.72)	0.83 (1.38)
Follow-up	0.75 (1.85)	0.91 (3.07)	0.27 (0.59)	0.71 (1.57)	0.33 (0.72)	0.28 (0.57)	1.00 (2.26)
Patients with outpatient visit, n (%)							
Baseline	4809 (99.86)	773 (100)	15 (100)	3200 (99.41)	14 (93.33)	18 (100)	54 (100)
Follow-up	4786 (99.38)	767 (99.22)	15 (100)	3217 (99.94)	15 (100)	18 (100)	54 (100)
Outpatient visits per patient, mean (SD)							
Baseline	17.07 (12.90)	17.94 (13.96)	34.33 (25.75)	17.37 (12.87)	20.53 (14.35)	25.50 (14.94)	25.15 (20.38)
Follow-up	17.14 (14.13)	19.06 (15.31)	32.20 (19.81)	21.59 (13.64)	30.53 (24.97)	26.78 (18.81)	27.02 (19.39)

[a]Total medical costs also included a small amount of costs from healthcare settings such as home healthcare, hospice facility, skilled nursing facility, etc.; these are not reported separately.

[b]Likely vedolizumab use was identified by prescription claims for vedolizumab, claims with unclassified HCPCS code J3590 along with a primary diagnosis code for UC or CD, or claims with HCPCS codes C9026 and J3380.

CD: Crohn's disease; ED: emergency department; HCPCS: Healthcare Common Procedure Coding System; SD: standard deviation; UC: ulcerative colitis.

Supplementary Table 3 - continued. Baseline and Follow-up Healthcare Resource Utilization and Costs for Patients with Crohn's Disease Who Initiated a Biologic, 1-year Follow-up (intention-to-treat population)

Outpatient utilization comprised physician's office visits and outpatient hospital and clinic visits. Pharmacy costs included costs on medication dispensed at pharmacy and administered inside the healthcare facility. Total medical costs were predominantly the sum of inpatient, ED and outpatient costs.[a]

	Biologic						
	**Adalimumab n=4816**	**Certolizumab n=773**	**Golimumab n=15**	**Infliximab n=3219**	**Natalizumab n=15**	**Ustekinumab n=18**	**Vedolizumab[b] n=54**
**Costs, US$ 2015, mean (SD)**							
Inpatient hospitalization costs per patient							
Baseline	10 814.81 (42 845.99)	11 935.42 (33 965.47)	820.37 (3177.30)	11 601.31 (33 803.83)	7929.32 (16 132.91)	53 814.72 (169 394.09)	12 557.05 (26 020.52)
Follow-up	9961.19 (36 545.68)	13 079.40 (46 260.88)	4860.47 (14 267.18)	8465.77 (44 395.61)	0 (0)	0 (0)	21 373.41 (82 775.07)
ED visits costs per patient							
Baseline	1694.43 (4227.83)	1846.99 (4699.16)	1377.77 (3383.99)	1612.21 (3853.58)	526.67 (888.42)	1684.61 (2272.81)	1287.95 (2870.08)
Follow-up	1569.02 (5259.36)	1914.44 (10 702.05)	362.43 (834.04)	1296.51 (3552.26)	764.71 (1599.96)	697.91 (1672.68)	3015.21 (8932.12)
Outpatient costs per patient							
Baseline	7959.12 (11 641.89)	8691.25 (13 242.51)	9238.01 (6904.25)	15 557.48 (26 965.07)	12 323.36 (16 274.12)	15 565.61 (12 767.66)	14 374.22 (26 344.35)
Follow-up	6213.93 (9196.11)	6428.85 (7666.81)	9805.12 (7473.64)	10 809.60 (16 923.58)	11 872.05 (10 106.68)	7348.29 (6526.97)	25 995.61 (20 568.17)
Total medical costs per patient							
Baseline	22 494.60 (47 879.54)	24 768.14 (41 583.94)	13 948.31 (12 112.39)	30 874.36 (43 873.62)	22 969.35 (21 103.59)	77 122.75 (187 410.77)	29 502.57 (36 072.23)
Follow-up	19 347.76 (42 967.16)	23 358.00 (52 465.72)	16 309.73 (16 938.57)	22 255.57 (50 299.10)	16 103.70 (13 396.67)	9582.51 (7642.45)	54 953.09 (86 026.56)
Pharmacy costs per patient							
Baseline	4983.74 (6664.84)	5769.31 (9735.85)	8771.72 (8437.04)	4396.38 (11 572.79)	19 355.67 (18 779.12)	8290.85 (9505.37)	11 748.41 (25 688.04)
Follow-up	36 951.52 (16 163.94)	31 020.63 (16 819.53)	31 188.58 (20 362.18)	36 553.22 (33 618.15)	62 443.95 (44 237.70)	68 702.92 (43 997.30)	32 918.95 (34 376.52)
Medical and pharmacy costs per patient							
Baseline	27 478.34 (48 710.01)	30 537.44 (43 265.60)	22 720.03 (14 146.07)	35 270.73 (45 569.51)	42 325.01 (23 350.83)	85 413.59 (191 871.70)	41 250.98 (43 025.37)
Follow-up	56 299.28 (45 633.51)	54 378.63 (54 094.75)	47 498.31 (25 332.73)	58 808.80 (60 812.85)	78 547.65 (51 208.20)	78 285.44 (46 093.88)	87 872.04 (88 511.99)

[a]Total medical costs also included a small amount of costs from healthcare settings such as home healthcare, hospice facility, skilled nursing facility, etc.; these are not reported separately.

[b]Likely vedolizumab use was identified by prescription claims for vedolizumab, claims with unclassified HCPCS code J3590 along with a primary diagnosis code for UC or CD, or claims with HCPCS codes C9026 and J3380.

CD: Crohn's disease; ED: emergency department; HCPCS: Healthcare Common Procedure Coding System; SD: standard deviation; UC: ulcerative colitis.
